# Single-Cell Transcriptome Analysis Reveals Development-Specific Networks at Distinct Synchronized Antral Follicle Sizes in Sheep Oocytes

**DOI:** 10.3390/ijms25020910

**Published:** 2024-01-11

**Authors:** Yukun Song, Nan Zhang, Yu Zhang, Junlan Wang, Qi Lv, Jiaxin Zhang

**Affiliations:** Inner Mongolia Key Laboratory of Sheep & Goat Genetics Breeding and Reproduction, College of Animal Science, Inner Mongolia Agricultural University, Hohhot 010018, China; 13507412927@163.com (Y.S.);

**Keywords:** sheep oocytes, antral follicle, single-cell transcriptomics, molecular signatures, signaling pathways

## Abstract

The development of the ovarian antral follicle is a complex, highly regulated process. Oocytes orchestrate and coordinate the development of mammalian ovarian follicles, and the rate of follicular development is governed by a developmental program intrinsic to the oocyte. Characterizing oocyte signatures during this dynamic process is critical for understanding oocyte maturation and follicular development. Although the transcriptional signature of sheep oocytes matured in vitro and preovulatory oocytes have been previously described, the transcriptional changes of oocytes in antral follicles have not. Here, we used single-cell transcriptomics (SmartSeq2) to characterize sheep oocytes from small, medium, and large antral follicles. We characterized the transcriptomic landscape of sheep oocytes during antral follicle development, identifying unique features in the transcriptional atlas, stage-specific molecular signatures, oocyte-secreted factors, and transcription factor networks. Notably, we identified the specific expression of 222 genes in the LO, 8 and 6 genes that were stage-specific in the MO and SO, respectively. We also elucidated signaling pathways in each antral follicle size that may reflect oocyte quality and in vitro maturation competency. Additionally, we discovered key biological processes that drive the transition from small to large antral follicles, revealing hub genes involved in follicle recruitment and selection. Thus, our work provides a comprehensive characterization of the single-oocyte transcriptome, filling a gap in the mapping of the molecular landscape of sheep oogenesis. We also provide key insights into the transcriptional regulation of the critical sizes of antral follicular development, which is essential for understanding how the oocyte orchestrates follicular development.

## 1. Introduction

Ovarian folliculogenesis is the complex maturation of an ovarian follicle through the primordial, primary, secondary, antral, and preovulatory developmental stages [1]. This developmental complexity is reflected in the high level of synchronization among the different cell types within one follicle that together produce a competent mature oocyte [2]. The generation of healthy oocytes relies on the coordinated development of somatic and germ cells in the ovarian follicle, and the rate of follicular development is determined using a developmental program intrinsic to the oocyte [3]. Activated preantral follicles develop to the antral stage, where follicle growth increases rapidly. The granulosa cells facilitate the accumulation of follicular fluid into a central cavity known as an antrum, giving rise to growing antral follicles [4]. Antral follicles develop in a wave-like pattern during which one or more follicles, “selected” from a group of small follicles (2–3 mm), develop into dominant follicles ≥ 6 mm in diameter. However, in mono-ovulatory bovine and ovine species, only one follicle becomes dominant, and subordinate follicles regress or degenerate [5,6].

The late antral follicle stage, when the oocyte acquires developmental potential, is critical for oocyte maturation [7]. Thus, the quality of an oocyte depends on its follicle, and the follicular size correlates with developmental outcomes [8]. Oocytes from larger follicles are more likely to reach the blastocyst stage than those from small follicles [9], but the regulation of gene expression is important in determining oocyte quality [10]. Thus, the relationship between the follicle size and the oocyte transcriptomic landscape should be determined and correlated with developmental competence. The oocyte depends on stored maternal transcripts that play a key role in the development of oocyte meiotic competence during the growth phase in preantral and early antral follicles. In addition, transcripts involved in energy production and metabolism are present in oocytes [11]. Differences in transcripts in the antral follicle development sizes could explain subsequent oocyte maturation and identify biomarkers of oocyte development. Oocyte development requires continuous intercommunication using highly coordinated paracrine, autocrine, and endocrine regulators, as well as gap junctions [12]. These oocyte-specific factors (OSFs) also promote the proliferation of follicular somatic cells and steroidogenesis. Transcription factors (TFs) that are translocated from the cytoplasm to the nucleus mediate signaling and regulate the cellular phenotype [13], and the formation of follicles may be coordinated by a TF expressed by the oocyte. Thus, it is important to (1) identify oocyte-secreted TFs and other OSFs involved in oocyte development, (2) classify these regulatory factors by antral follicle size, and (3) describe the dynamic associations of key factors between follicles of different sizes.

Single-cell RNA sequencing (scRNA-seq) is a key tool for characterizing tissue heterogeneity at a cellular level [14], and it identifies changes in gene transcripts at the single-cell level that are undetectable using bulk RNA sequencing. Using scRNA-seq to efficiently reveal the gene regulation and expression changes of oocytes during antral follicle development is crucial for understanding the details of oocyte development. Most oocyte transcriptome studies use animal models [15]. The scRNA-seq transcriptomes of human oocytes at five follicular sizes revealed candidate secretory biomarkers of ovarian reserve [16]. The scRNA-seq transcriptome in germinal vesicles (GVs) and in in vitro metaphase II (MII) human oocytes identified maturation differences in oocytes [17]. However, the developmental pattern and transcriptional regulatory networks in sheep oocytes during antral follicle development differ from human and mouse oocytes [18]. Currently, transcriptomics has revealed the profile of oocyte RNA in different species, and several studies have revealed the high conservation of oocyte-specific genes involved in meiosis, mRNA binding, and metabolism in oocytes [19,20]. However, the expression patterns of germline genes (TGF-β superfamily members [21]; *NLRP*, *KHDC1/DPPA5* [22]; *BCAR4* [23]; *ZP* [24]) are reported to display considerable interspecies variations. Overall, these differences remind us that it is important to conduct studies of transcriptional regulatory networks within diverse mammalian animal oocytes. 

Here, we analyzed the transcriptome of single oocytes from antral follicles of different sizes to identify dynamic changes in gene expression throughout antral follicle growth. We identified unique features of the transcriptional machinery associated with developmental-specific expression patterns. We also identified stage-specific gene signatures in sheep oocytes, including oocyte-specific genes, oocyte-secreted factors, and TFs, which could provide oocyte development-specific profiles. We found differences in transcripts associated with specific biological processes and key signaling pathways that were associated with antral follicle development, providing information on expression patterns of hub genes regulating oocyte maturation. Thus, our characterization of the transcriptional landscape of antral follicle oocytes provides insights into the regulation of follicle growth. Specifically, distinctive markers exclusive to particular developmental sizes were unveiled, presenting themselves as prospective novel biomarkers and plausible targets for therapeutic intervention within the human ovary.

## 2. Results

### 2.1. Overview of Single-Oocyte Sequence Data

**Figure 1:**
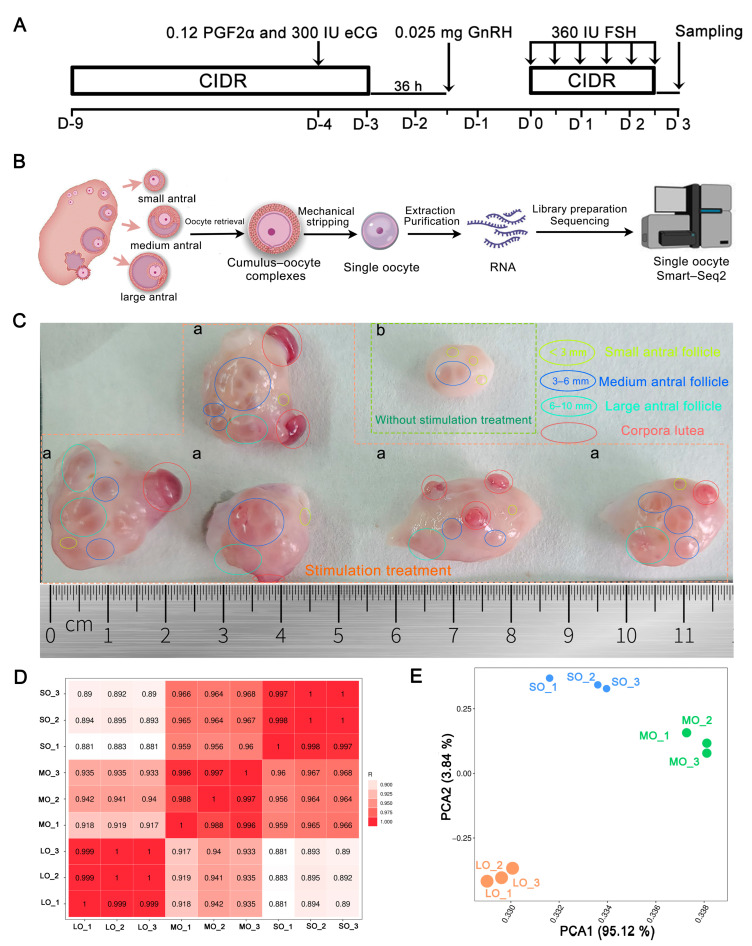
Single-cell RNA-seq transcriptome patterns in sheep oocytes: (**A**) Estrus synchronization and superovulation treatment schedule. On day 0, an intravaginal progesterone device was inserted, and ovarian stimulation was performed using multiple decreasing doses of FSH. PGF2α, prostaglandin F2α; eCG, equine chorionic gonadotropin; GnRH, gonadotrophin-releasing hormone; FSH, follicle-stimulating hormone; LOPU, laparoscopic ovum pick-up. (**B**) Flowchart for sheep oocyte scRNA-seq. Cumulus–oocyte complexes (COCs) were recovered from each sheep and classified according to antral follicle size: small (<3 mm), medium (3–6 mm), and large (6–10 mm). The oocytes and granulosa cells (GCs) were stripped mechanically, and oocyte transcriptomes were compared using single-cell RNA sequencing analysis (Smart-seq2). (**C**) Representative image of sheep ovaries (a) with or (b) without stimulation treatment. Small, medium, and large antral follicles are depicted in yellow, blue, and green circles, respectively, whereas red circles highlight the corpus luteum. (**D**) Heatmap with color highlighting the distance matrix (Pearson correlation). The color gradient ranges from dark red (minimum distance) to light red. Horizontal bars represent the antral follicle phase. LO, oocyte from large antral follicle; MO, oocyte from medium antral follicle; SO, oocyte from small antral follicle. (**E**) Principal component analysis (PCA) of the transcriptomes from all oocytes. Purple indicates SO, blue indicates MO, and green indicates LO.

To identify changes in oocyte transcripts at different antral follicle sizes, we collected oocytes from antral follicles at different developmental sizes, including small (≤3 mm, SO1, SO2, SO3), medium (3–6 mm, MO1, MO2, MO3) and large (6–10 mm, LO1, LO2, LO3) antral follicles for single-oocyte RNA-seq using the Smart-seq2 protocol (Figure 1A–C and Table S1). Following the library construction and deep sequencing of oocyte transcriptomes, we obtained a total of 372,027,250 bases from the nine sequence libraries, producing a total of 334,979,082 (90.06%) clean reads (Figure S1A and Table S2). The mapping efficiency of the clean reads to the sheep genome was high, with an average of 97.55% for the small antral oocytes (SOs), 97.15% for the medium antral oocytes (MOs), and 96.68% for the large antral oocytes (LOs), and a unique mapped rate average of 91.22% (Figure S1B). The quality of these mRNA libraries was high enough to use them for statistical analyses. We identified an average of 15,341 expressed genes per oocyte, with a total of 138,073 expressed genes; 14,512 were expressed in the SO group, 15,300 in the MO group, and 16,212 in the LO group; however, the LO group had the most mRNAs. We found that RNA synthesis was still detectable in the period following oocyte growth arrest and germinal vesicle breakdown (GVBD), and transcription ceased only at GVBD. Therefore, it is possible to resolve differences in mRNA expression levels between sizes and identify the factors involved in oocyte developmental competence.

Using principal component analysis (PCA) for the groups of oocytes, we observed three distinct subpopulations, which corresponded to the morphological classification of antral follicular development (Figure 1D,E). Thus, antral follicles at different developmental sizes exhibited different gene expression dynamics, as evidenced by the distinct transcriptomes.

### 2.2. Gene Expression Dynamics and Transcriptional Characteristics of Oocytes during Antral Follicle Development

**Figure 2:**
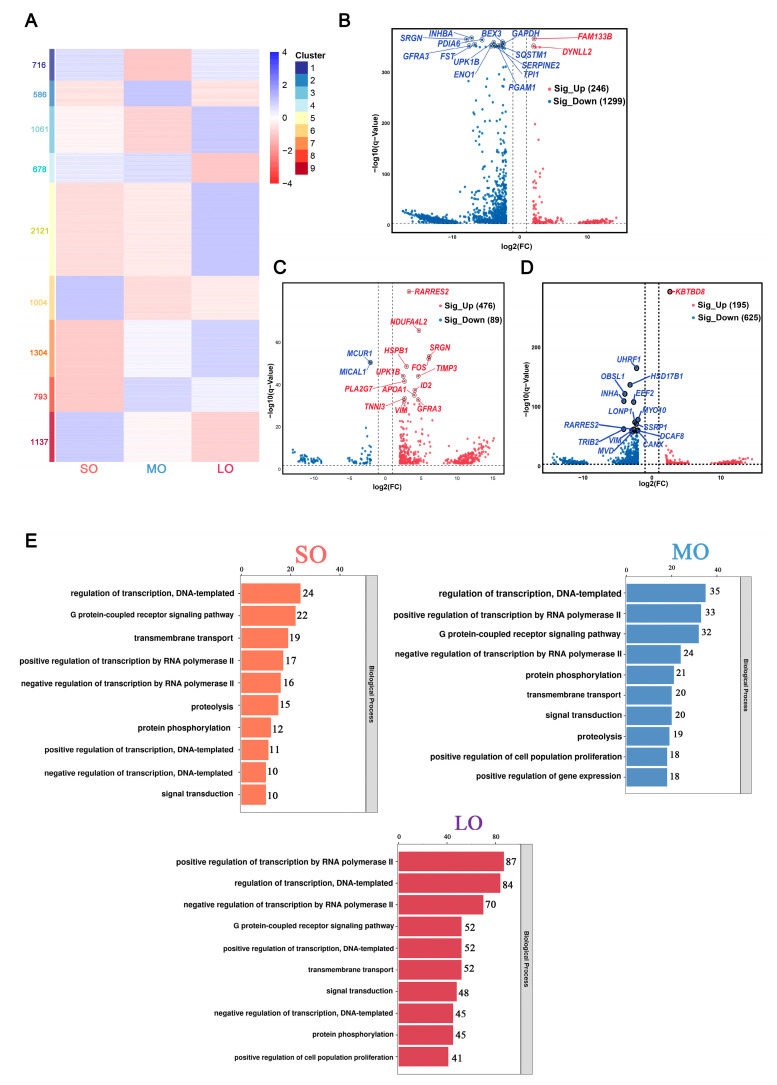
Gene expression dynamics and transcriptional characteristics of oocytes: (**A**) Heatmap of 9400 differentially expressed genes (DEGs) in oocytes at three sizes of antral follicle development. The number of DEGs is indicated on the y-axis and the sizes of follicular development on the x-axis. The color key indicates the relative gene expression from blue (high) to red (low). (**B**–**D**) Volcano plots showing DEGs for oocytes in different groups: red, upregulated DEGs; blue, downregulated DEGs (log_2_ (FC) > |2| and FDR < 0.05). (**E**) Significantly enriched GO terms (biological processes) for DEGs in the oocytes at three antral follicle sizes. The top 10 terms for each size are based on the normalized enrichment score (NES). FDR < 0.05.

To determine the global expression profiles during antral follicle development, we characterized the 9400 differentially expressed genes (DEGs) for the sizes of follicular development in oocytes (false discovery rate [FDR, *q*-value] < 0.05; Figure 2A and Figure S2A,B). To characterize gene expression dynamics between consecutive stages in antral follicle development, we compared DEGs and found 2141 genes overrepresented in the SO group, 2058 genes overrepresented in the MO group, and 4486 genes overrepresented in the LO group. An unsupervised cluster analysis identified nine clusters of genes according to their expression patterns. The genes in cluster 5 (2122) and cluster 7 (1304) were under-expressed in the SO group and were upregulated during antral follicular development, with a peak expression in the LO group, suggesting that they indicate oocyte maturation. However, the transcripts for genes in cluster 9 (1137) and cluster 6 (1004) accumulated before follicle selection and dominance, suggesting that they represent the resting follicle pool. The gene expression patterns of cluster 2 (586), cluster 8 (792), and cluster 4 (678) showed expression in the MO group (Figure S2C). However, because the oocyte mRNA increased substantially during antral follicular development, the putative DEGs might simply reflect changes in the relative abundance of transcripts, with only some of them corresponding to changes in transcriptional state.

To identify transcripts specifically enriched at each developmental stage from the DEG expression profiles, we considered those genes with a log_2_ transformed fold change (log_2_FC) > |2| and FDR < 0.05 as differentially expressed genes. This yielded 1299 genes overrepresented in the LO, 246 genes overrepresented in the SO (Figure 2B), and 565 genes identified in a comparison between the MO and SO groups (Figure 2C). Compared to the SO group, 476 genes were upregulated in the MO group, which accounted for the majority of DEGs, and only 89 genes were downregulated. Using the same criteria, we screened 820 genes, including 195 upregulated genes and 625 downregulated genes in the MO vs. LO oocytes (Figure 2D). The top 10 upregulated or downregulated genes in the SO vs. LO, MO vs. SO, and MO vs. LO groups are listed in Table S3. Using gene ontology (GO) and Kyoto Encyclopedia of Genes and Genomes (KEGG) analysis of DEGs in oocytes at different sizes to characterize their function in antral follicle growth, we identified several significantly enriched biological processes associated with each size of follicular development (Figure 2E and Figure S2D–F). 

### 2.3. Gene Expression Signatures of Oocytes at Different Antral Follicle Sizes

**Figure 3:**
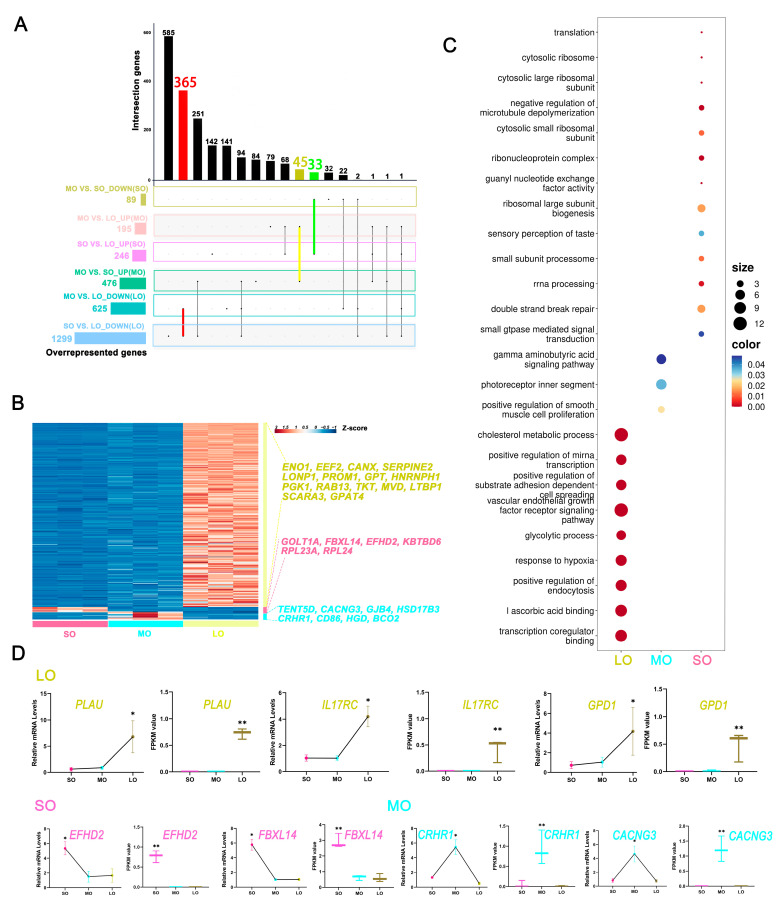
Dynamic gene expression signatures of oocytes at three antral follicle sizes: (**A**) Upset plot showing the number of overlapping upregulated and downregulated DEGs in each group. The histogram represents the number of co-expressed genes. (**B**) Heatmap showing gene expression levels in different sizes. The number of DEGs is indicated on the y-axis and the sizes of follicular development on the x-axis. Right: representative stage-specific DEGs are shown. The three clusters of genes represent their functions. (**C**) Representative GO terms enriched for SOs, MOs, and LOs. The top 15 terms for each size are based on the normalized enrichment score (NES). FDR < 0.05. (**D**) Expression levels of stage-specific gene (*PLAU*, *IL17RC*, *GPD1*, *EFHD2*, *FBXL14*, *CRHR1*, and *CACNG3*) using qPCR (mRNA level) and Smart-seq2 (FPKM value) in SOs, MOs, and LOs. Box-plots indicate the mean ± standard error (*n*  =  3); two-tailed *t-*test *p*-values are indicated (* *p* < 0.05, ** *p* < 0.01).

Using Venn diagrams, we identified 365 genes that overlapped the 625 genes (MO vs. LO) and 1299 genes (SO vs. LO) in the LO group (Figure 3A), and 45 genes overlapped in the MO group, whereas 33 genes overlapped in the SO group. Surprisingly, there was no overlap in the three groups for upregulated genes. We clustered the overlapping genes with DEGs (SO vs. MO vs. LO; 9400) and considered those genes with FDR < 0.05 as the stage-specific genes. We identified 222 stage-specific genes, including genes expressed in the oocytes from each antral follicular size (Figure 3B and Table S4). Most of these genes (208) were upregulated during antral follicular development, with the highest expression in the large antral follicle. In addition, eight and six genes were highly expressed in the MO and SO groups, respectively.

We used GO analysis of genes specific to particular oocyte sizes to determine the regulatory functions involved in antral follicle development (Figure 3C). The transcription coregulator binding (representative genes *ESR1*, *JUND*, *MYC*, *NR5A2*, and *SMARCD3*), glycolytic process (representative genes *IER3*, *PFKL*, *PFKP*, *PGK1*, and *ENO1*), vascular endothelial growth factor receptor signaling pathway (representative genes *BCAR1*, *FGF10*, *FLT4*, *NRP1*, *PTK2B*, *VEGFA*, and *VEGFC*), positive regulation of miRNA transcription (representative genes *AR*, *JUN*, *MYC*, and *NGFR*), response to hypoxia (*DDIT4*, *EPO*, *HIF1A*, *LONP1*, *NOS2*, *P2RX2*, *PLAU*, *PLOD1*, *PLOD2*, *SOD3*, and *VEGFA*), and cholesterol metabolic process (representative genes *CH25H*, *CLN8*, *CYP11A1*, *LCAT*, and *SMPD1*) were enriched in the LO group, and GO enrichment items with FDR < 0.05 are shown in Figure 3D. Interestingly, the regulation of calcium ion import (representative genes *CACNG3*), gap junction channel activity (representative genes *GJB4*), and steroid biosynthesis (representative genes *HSD17B3*) were enriched in the MO. We also identified candidate biomarkers uniquely expressed in the medium antral follicle: *TENT5D*, *HGD*, *CRHR1*, and *CD86*, which are involved in mRNA stabilization [25], tyrosine or L-phenylalanine metabolism [26], the activation of G protein-coupled receptors [27], and adaptive immunity [28], respectively. In addition, ribosome biogenesis was enriched in the SO group, with increased expression of ribosome-related genes (representative genes *RPL23A* and *RPL24*). *GOLT1A* plays a critical role in vesicle-mediated protein transport from the endoplasmic reticulum to the Golgi [29]. KBTBD6, as part of the Cul3 (KBTBD6/7)-RING ubiquitin ligase complex [30], is involved in the proteasome-mediated ubiquitin-dependent protein catabolic process and regulates RAC1 signal transduction and downstream biological processes, including the organization of the cytoskeleton, cell migration, and cell proliferation [31]. Members of the F-box protein family, such as *FBXL14*, are newly recognized E3 ligases that play a critical role in ubiquitylation [32]. *EFHD2* is a conserved calcium-binding protein that plays a role in vitro in calcium signaling, apoptosis, the actin cytoskeleton, and the regulation of synapse formation [33]. Stage-specific gene expression profiles and qPCR revealed a core hub of genes that regulate specific functions at each size of oocyte development, including *EFHD2* and *FBXL14* in the SO group; *CRHR1* and *CACNG3* in the MO group; and *PLAU*, *IL17RC*, and *GPD1* in the LO group (Figure 3D). These signature genes may serve as potential cell-specific markers for each follicle size. In addition, the subsets of 222 stage-specific signature genes in oocytes could serve as markers of oocyte developmental competency. Together, our results reveal the atlas of stage-specific transcriptional regulatory networks underlying antral follicle development in sheep.

### 2.4. Characterization of Key Pathways throughout Antral Follicle Development

Gene Set Enrichment Analysis (GSEA) and KEGG analysis were used for pairwise comparisons of the follicular sizes to identify changes in signaling pathways during the transition from the small to the medium size during follicle recruitment (FDR < 0.05). Ribosome and phosphatidylinositol signaling systems were upregulated in the SO group. In contrast, 22 pathways were enriched in the MO group, including ovarian steroidogenesis, fat digestion and absorption, glutathione metabolism, and neuroactive ligand–receptor interaction (Figure S3A). Notably, follicle selection is a critical step in oocyte maturation. We identified 25 functional pathways in the LO group (FDR < 0.05), including glycolysis/gluconeogenesis, the pentose phosphate pathway, the biosynthesis of amino acids, apoptosis, and cellular senescence (Figure S3B). In addition, the nicotine addiction pathway and neuroactive ligand–receptor interaction were overrepresented in the MO group. These intra-follicular metabolic signaling pathways likely mediate the transition from the medium to the large antral follicular size, which involves the coordinated interaction of oocytes and granulosa cells for follicular development. Finally, the greatest increase in the number of signaling pathways occurred during the transition of oocytes from small to large antral follicles. Overrepresented pathways in the LO group included ribosome, oxidative phosphorylation, aldosterone-regulated sodium reabsorption, and thermogenesis (Figure S3C).

**Figure 4:**
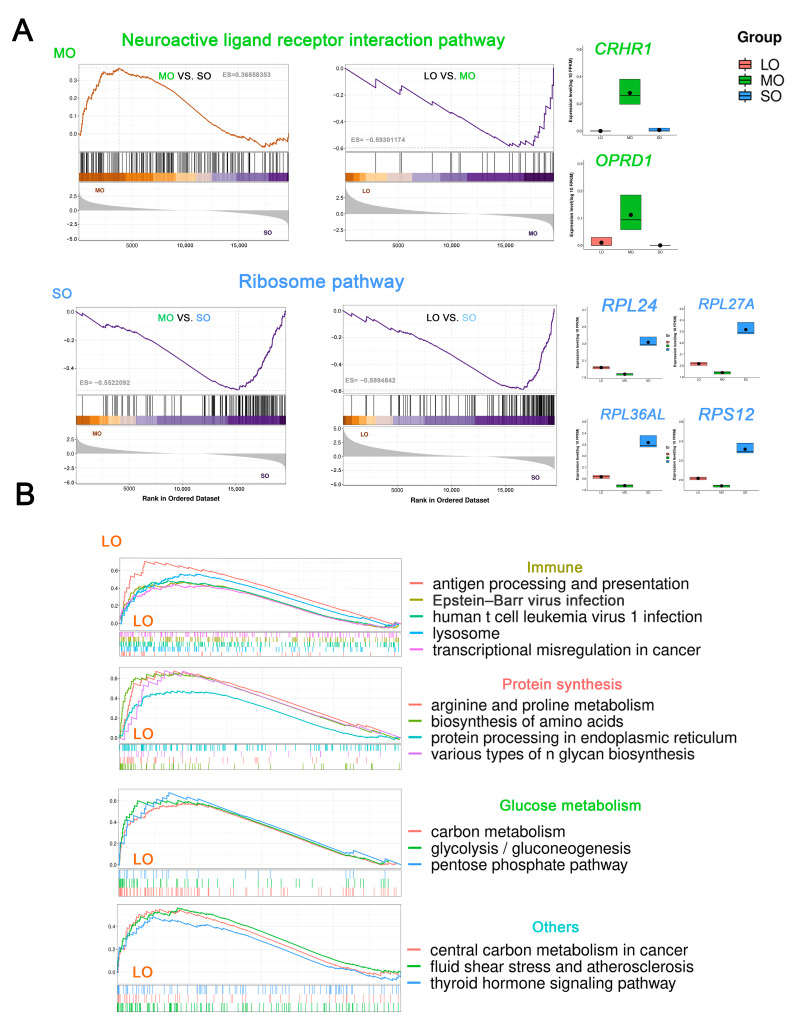
Signaling pathways enriched at each antral follicle size: (**A**) Left: GSEA enrichment plots of KEGG signaling pathways. Right: boxplots of key component genes in oocytes from the medium and small antral follicle. (**B**) Left: GSEA enrichment plots of KEGG signaling pathways. Right: boxplots of key component genes in oocytes from large antral follicles.

To better understand whether these signaling pathways were enriched in sheep oocytes at specific antral follicle sizes, we merged or overlapped the results described above to identify pathways that were specific or homogeneous during antral follicular development (Figure 4A). Ribosome genes were expressed predominantly in the SO group with a high expression of several ribosomal protein genes (representative genes *RPL24*, *RPL27A*, *RPL36AL*, and *RPS12*). Interestingly, the neuroactive ligand–receptor interaction signaling pathway (representative genes *CRHR1* and *OPRD1*) was highly expressed in the MO group. In addition, we identified 15 co-expressed signaling pathways in the LO group (Figure 4B), and, among these, the biosynthesis of amino acids and various types of N-glycan, arginine, and proline metabolism, and protein processing in the endoplasmic reticulum play a key role in oocyte maturation. Due to the critical role of glycans in protein modification and the regulation of diverse cellular functions [34], we further analyzed the N-glycan biosynthesis pathway and found 12 highly expressed genes, including *MGAT4B* [35], *ALG3* [36], and *DDOST* [37], suggesting that N-glycosylation was one of the key factors affecting sheep oocyte maturation.

### 2.5. Expression Patterns of Oocyte-Specific Genes at Antral Follicle Developmental Sizes

**Figure 5:**
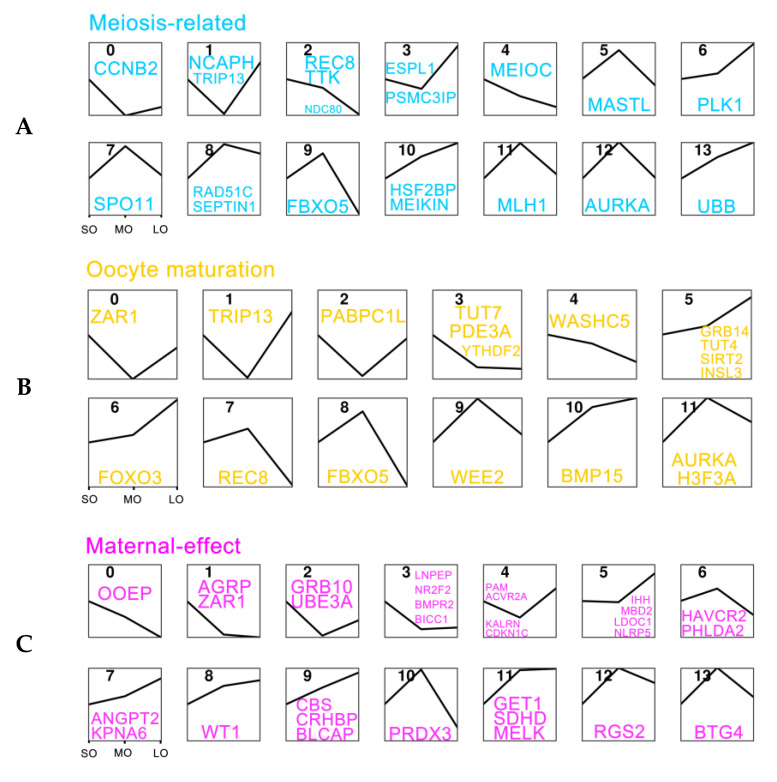
Expression patterns of oocyte-specific genes: (**A**) Classification of meiosis-related gene expression clusters. The sizes of antral follicular development are presented on the x-axis. The y-axis shows the relative expression for each cluster; (**B**) Classification of oocyte maturation gene expression clusters; (**C**) Classification of maternal-effect gene expression clusters.

We integrated our sheep oocyte transcriptomes with the human and mouse transcriptomes [38] to characterize the global expression profiles of oocyte-specific genes. To study the expression patterns of meiosis-related genes in oocytes, we obtained 38 genes involved in female meiosis I and nine genes involved in female meiosis II from the Human Protein Atlas (HPA) database, and found 23 meiosis I genes and six meiosis II genes (FDR < 0.05) that were differentially expressed across different sizes of oocyte maturation (Table S5). Based on the expression patterns, an unsupervised cluster analysis identified 14 clusters of genes (Figure 5A). Most of these genes (including *ESPL1*, *PSMC31P*, *PLK1*, *HSF2BP*, *MEIKIN*, and *UBB*) showed upregulation as antral follicular development proceeded, with the highest expression at the large antral follicle. An analysis of 44 genes from the Mouse Genome Informatics (MGI) and HPA databases involved in oocyte maturation revealed that some of these genes (including *GRB14*, *TUT4*, *SIRT2*, *INSL3*, *FOXO3*, and *BMP15*) increased gradually during oocyte development whereas others (such as *REC8*, *FBXO5*, *WEE2*, *AURKA*, and *H3F3A*) were expressed at high levels at the medium antral follicle (Figure 5B and Table S6). Among 62 maternal-effect genes, which play a critical role in mammalian development before zygote genome activation [39], 38 genes were expressed during antral follicle development (Table S7). The genes in cluster 5 (*IHH*, *MBD2*, *LDOC11*, and *NLRP5*), cluster 7 (*ANGPT2* and *KPNA6*), cluster 8 (*WT1*), and cluster 11 (*GET1*, *SDHD*, and *MELK*) were upregulated during follicular maturation (Figure 5C). However, the genes in cluster 6 (*HAVCR2* and *PHLDA2*), cluster 10 (*PRDX3*), cluster 12 (*RGS2*), and cluster 13 (*BTG4*) were expressed before follicle selection. The genes *OOEP*, *AGRP*, *ZAR1*, *GRB10*, *UBE3A*, *LNPEP*, *NR2F2*, *BMPR2*, and *BICC1* were under-expressed in oocytes at all sizes of follicular development. Among the maternal-to-zygotic transition genes, *BTG4* and *PABPN1L* were relatively highly expressed, increasing in abundance during oocyte development (Table S8).

### 2.6. Identification of Oocyte-Secreted Factors throughout Antral Follicle Development

**Figure 6:**
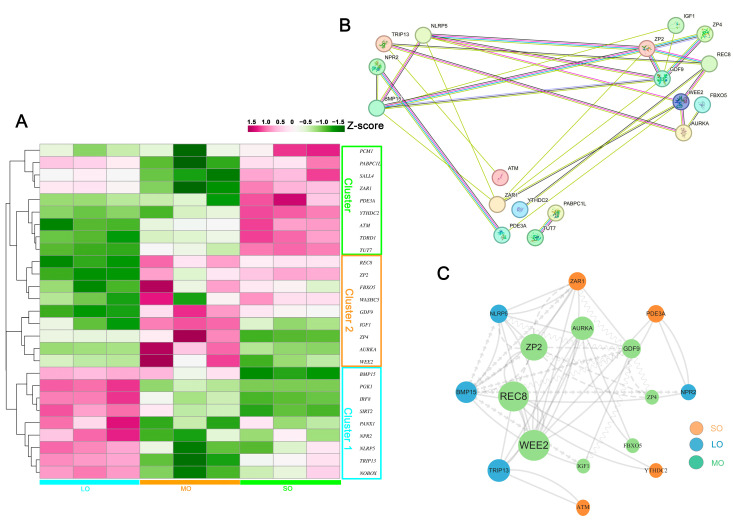
Oocyte-secreted factors at three antral follicle sizes: (**A**) Heatmap of DEGs for oocyte-secreted factors (OSFs). The genes are shown on the y-axis according to the three clusters based on the similarity of expression patterns, and the sizes of antral follicular development are shown on the x-axis. The color key indicates relative gene expression from green (low) to purple (high). (**B**) Analysis of the protein–protein interaction (PPI) network of OSFs for each follicle size. (**C**) Visualization of the gene network of OSFs using CytoScape. The 16 genes with the highest connectivity within the network are charted, and hub genes within the network are shown by the size of the dots. The nodes represent proteins, and the edges represent the interaction.

We identified 38 genes encoding sheep oocyte secretory proteins that were differentially expressed during antral follicular development (Table S9). The expression patterns of the genes from each cluster and their functions are shown in Figure 6A and Figure S4A. Cluster 1 comprised nine genes expressed only in the LO group, suggesting their involvement in the subsequent developmental competence of oocytes. Cluster 2 included nine genes expressed only in the MO group, suggesting their involvement in the initiation of follicle selection. Cluster 3 consisted of nine genes that represented follicle recruitment. A protein–protein interaction (PPI) analysis of the oocyte-specific protein-coding genes showed that the PPI network was reconstructed from 18 gene symbols (Table S10). Among these, secretory factors GDF9, BMP15, NLRP5, ZP2, ZAR1, and REC8 formed the core of the PPI network (Figure 6B,C; Table S11). These genes were related to oocyte development and were involved in progesterone-mediated oocyte maturation, the HIF-1 signaling pathway, and cytokine–cytokine receptor interactions. These oocyte-specific secretory factors reveal the diverse functions of oocytes in antral follicle development. Two important oocyte-secreted factors are growth differentiation factor 9 (GDF9) and bone morphogenetic protein 15 (BMP15). They activate signaling pathways in cumulus cells (CCs) that regulate the differentiation and the distinctive phenotype of CCs. Furthermore, the interaction of GDF9 and BMP15 with follicle-stimulating hormone (FSH) potentiates the expression of IGF-2 in human granulosa cells [40]. *NLRP5* is a maternal lethal effect gene in mammals, and the loss of NLRP5 in mouse oocytes triggers premature activation of the mitochondrial pool, causing mitochondrial damage and apoptosis [41]. The zona pellucida surrounding all mammalian oocytes plays a vital role in fertilization and early development. Knockout of zona pellucida protein ZP2 in female mice produces oocytes without a ZP, resulting in fertilization failure and infertility [42]. Zygote arrest 1 (*ZAR1*) is the first oocyte-specific maternal-effect gene known to function in the oocyte-to-embryo transition [43]. Rec8-containing cohesin, bound to Smc3/Smc1a or Smc3/Smc1b, maintains the bivalent cohesion in mammalian meiosis [44]. Sister chromatid cohesion mediated by the cohesin complex is essential for chromosome segregation in mitosis and meiosis [45]. Here, we found that the *REC8* transcript decreased in oocytes from large antral follicles, which may indicate oocyte meiotic resumption. In addition, Wee1-like protein kinase 2 (*WEE2*), a key regulator of meiosis during prophase I, is involved in maintaining meiotic arrest [46]. Further evaluation of the expression levels of OSFs in the follicular fluid may identify novel biomarkers for assessing oocyte development.

### 2.7. TF Regulatory Networks in the Oocytes

**Figure 7:**
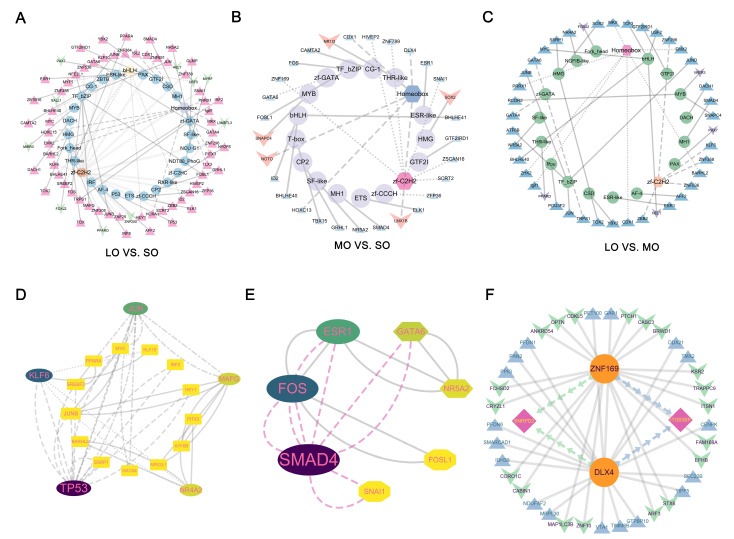
Mapping TF regulatory networks in oocytes from three antral follicle sizes: (**A**–**C**) Visualization using CytoScape of the TFs and TF family regulatory networks at the transition of each size of antral follicle development. The inner circle shows the TF family, whereas the traits indicate the number of TFs contained. An upward triangle indicates a significantly upregulated TF and an inverted triangle indicates a significantly downregulated TF. (**D**) The PPI network of the 18 TFs in oocytes from the large antral follicle size was constructed using the STRING database. The nodes represent proteins, and the edges represent the interactions. Outer circles represent hub TFs, and different sizes indicate criticality. (**E**) The PPI network of the seven TFs co-expressed in small and medium antral follicles was constructed using the STRING database. The nodes represent proteins, and the edges represent the interactions. (**F**) CytoScape plots of the TF–DEG regulatory networks in medium antral follicles. Yellow circles represent TFs, and triangles and diamonds represent co-regulated target genes.

To construct regulatory networks of TFs for the SO and LO groups, we obtained all 1376 sheep TFs from the Animal Transcription Factor Database (TFDB v4.0), a comprehensive database for predicting and classifying genome-wide TFs. During the development from the small to large antral sizes, we found 73 differentially expressed TF genes (Figure 7A); 63 TF genes were highly expressed in the LO, and the others were highly expressed in the SO. This suggests that these TFs play a critical role in the follicular cavity and oocyte maturation. During the transition from the small to the medium antral size, 24 TF genes were upregulated, and five TF genes (*NOTO*, *NR1I3*, *LMX1B*, *SOX2*, and *SNAPC4*) were downregulated (Figure 7B). Genes for 37 TFs were overexpressed in the LO group vs. the MO group and, thus, are candidate regulators of the medium-to-large antral transition. The genes *MIER3*, *PAX7*, *HELT*, *PPARD*, and *FOXJ2* were more highly expressed in the MO group (Figure 7C). To identify the master regulators of sheep antral follicle development, we overlapped DEGs for TFs at different sizes (Figure S5A). A large number of TFs were expressed only in the LO group, indicating possible regulatory roles in the cytoplasmic and nuclear maturation of oocytes (Figure S5B). Searching for recurring instances of neighboring TFs with STRING provided a predicted PPI network for 50 TFs as hub genes for the LO. The cellular tumor antigen p53 (*TP53*) TF, along with Kruppel-like factor 6 (*KLF6*) and Jun proto-oncogene *(JUN*), are hub genes for the positive regulation of RNA polymerase genes (Figure 7D and Figure S5D; Table S12). Among the 21 TFs that were co-expressed in the MO and LO groups (Figure 7E and Figure S5E; Table S13), the gene for mothers against decapentaplegic homolog (*SMAD4*), the central mediator of TGF-β signaling, is indispensable for oocyte formation and development [47].

Next, we speculate that *L3MBTL3*, *MYRF*, *SALL1*, *USF3*, *NOTO*, *NR1I3*, *LMX1B*, and *ZNF200* most likely initiate the transcription network in small antral follicles, whereas *DLX4*, *ZNF169*, and *TBX15* could be the unique regulators of follicle selection. To determine the potential roles of TFs in regulating the expression of DEGs, a TF–gene regulatory network was established based on predicted stage-specific target genes. In the SO group, key TFs (*MYRF*, *SALL1*, and *LMX1B*) showed a significant correlation with multiple DEGs (Figure S5C). Alkylglycerone phosphate synthase (*AGPS*), nuclear import carrier for Hsp70s (*HIKESHI*), and ectodermal-neural cortex 1 (*ENC1*) encode proteins that promote oocyte stability through lipid metabolism [48], protein transport [49], and the cytoskeleton [50], respectively. In addition, we identified significant TF target gene pairs of *ZNF169*–*ITGB3BP*, *ZNP169*–*SNRPD2*, *DLX4*–*ITGB3BP*, and *DLX4*–*SNRPD2,* which may play a role in mRNA processing at the medium antral follicle size (Figure 7F). 

Furthermore, utilizing publicly available databases (hTFtarget, ENCODE [51], and GTEx_Ovary: https://gtexportal.org/home/tissue/Ovary?tissueSelect=Ovary, accessed on 10 July 2023), we predicted the upstream regulatory factors linked to candidate transcription factors at different antral follicle sizes. Our investigation revealed 66, 33, and 14 regulatory factors corresponding to the LO, MO, and SO groups, respectively (Tables S14–S16). Subsequently, these regulatory factors were paired with stage-specific genes. Our findings unveiled four specific upstream regulators—*JUND*, *MYC*, *BHLHE40*, and *JUN*—coordinating the expression of candidate transcription factors *TP53* and *KLF6* during the LO (Figure S5F–H). A further characterization of these TFs will provide insights into the transcriptional control of oocyte-induced antral follicle development.

## 3. Discussion

Understanding the mechanisms underlying oocyte maturation is essential for understanding reproductive biology. From the GV stage, oocytes reduce transcription and rely on stored mRNA and the translational machinery to control gene expression [52]. Thus, to understand oocyte maturation, it is important to determine the transcriptional profile of genes that regulate this process. In this study, we removed the oocytes from single follicles and generated scRNA-seq data from oocytes at the small, medium, and large antral follicle sizes for a comprehensive assessment of gene expression. We identified DEGs using pairwise comparisons of the three sizes and found that the LO group had more specifically expressed genes than the SO and MO groups. The oocyte is transcriptionally active when the antral follicle first forms, but stored transcripts are critical for the initial stages of embryonic development until transcription is activated. A total of 565 DEGs were found in the MO vs. SO groups, representing the protein transport, glycolytic processes, and regulation of mRNA stability. Circulating FSH supports the growth of a group of antral follicles until the largest follicle produces estradiol, which occurs in sheep when the largest follicle is ~6 mm. GO analysis revealed that the biological processes that were enriched in the LO vs. the MO group included the regulation of transcription, positive regulation of fibroblast proliferation, and response to hypoxia, which may indicate intrinsic differences in these follicle sizes. These signature genes may serve as cell-specific markers for each follicle size. Furthermore, the *EFHD2* and *FBXL14* genes in oocytes from the SO group, *CRHR1* and *CACNG3* in oocytes from the MO group, and the top 15 DEGs in oocytes from the LO group could serve as markers of oocyte maturation and developmental competency and may provide essential genetic tools for stage-specific labeling.

Although the transition from the small to the large antral is a key step in oocyte development, the molecular mechanisms and signaling pathways that drive this transition are unknown. Here, we preliminarily identified signaling pathways associated with oocyte development and maturation based on the expression patterns of maternal transcripts and genes for key pathways. We found that ribosome biogenesis was regulated in the small antral follicle, producing sufficient ribosomes to support early embryo development until the initiation of zygotic transcription [53]. Thus, the oocyte translation program plays a critical role in the preparation for oocyte development. Oocyte–granulosa cell communication, mediated by several ligand–receptor pairs, is essential for oocyte development [54], and ligand–receptor pairs transmit signals between granulosa cells and oocytes to coordinate oocyte–granulosa cell functions (e.g., the regulation of transcription, translation, apoptosis, cell differentiation, and transport). Combined with other studies, we reasoned that signaling between the oocyte and CCs may be regulated by the neuroactive ligand–receptor interactions pathway [54]. Moreover, the key active pathways in large antral follicles are primarily in protein synthesis and glucose metabolism, although we also found that the thyroid hormone signaling pathway was upregulated in the oocytes from large antral follicles. Interestingly, thyroid hormones may directly affect oocytes, as specific binding sites for thyroxin are found in mouse and human oocytes. The thyroid hormone status may also affect the estrogenic sensitivity of the ovary [55], which is required for follicular dominance. Our results indicate that regulation of mRNA translation is important in follicle recruitment and that the neuroactive ligand–receptor interaction pathway is critical for the activation of oocyte maturation.

Oocyte quality determines the ability of the oocyte to overcome meiotic arrest and progress to the MII stage [56]. Thus, we identified the gene expression signatures for meiosis-related and oocyte maturation-associated genes and found that *MLH1* and *AURKA*, which are associated with spindle building and chromosome segregation [57], were among the genes highly expressed in medium antral follicles. Lodde et al. [58] demonstrated a temporal relationship between chromatin remodeling and the main morpho-functional events that characterize the final growth phase in bovine oocytes. *MEIOC*, together with *YTHDC2*, promotes a meiotic cell cycle program via post-transcriptional control [59]. In addition, changes in the expression of maternal-effect genes revealed different hub genes at each oocyte stage. The maternal-effect genes, which are the mRNAs produced and stored in the cytoplasm of growing oocytes [60], are the sole substrates for protein synthesis after transcription is silenced in oocytes. The transcript levels of *ANGPT2*, *KPNA6*, *CBS*, *CRHBP*, and *BLCAP* decreased sharply with antral follicle development. CBS is required for the acetylation of α-tubulin for proper spindle assembly during meiotic maturation, which affects oocyte quality [61]. Here, we provide evidence that meiotic and developmental competence is acquired gradually during the antral follicular process. This involves sequential events regulated by maternal transcripts within the oocyte, including transcriptional regulation, spindle assembly, and chromosomal recombination. Our high-quality data reveal extensive changes in gene expression associated with oocyte development at the single-cell level.

OSFs are critical for the regulation of oocyte maturation and follicular development, and the regulation of the ovarian follicular microenvironment through OSFs may be important for oocyte development. Two oocyte-specific growth factors, growth differentiation factor 9 (GDF9) and bone morphogenetic protein 15 (BMP15), are critical players in the follicular development of sheep [62]. Interestingly, we found expression of the gene for natriuretic peptide receptor (*NPR2*) in the MO group. *NPR2* in sheep, unlike in mice and pigs, is not only in CCs but is also in oocyte membranes, indicating that C-type natriuretic peptide (CNP) may activate intra-oocyte cGMP production via the membrane *NPR2* in addition to the CC-mediated pathway [63]. In meiosis, Aurora kinase A (*AURKA*) regulates spindle building and chromosome segregation in oocytes [64], whereas thyroid hormone receptor interactor 13 (*TRIP13*), which is involved in recombination, is required early in meiotic recombination [65]. We showed here that meiotic resumption is activated at the medium antral follicle, whereas chromosome alignment and segregation occur at the large antral follicle, suggesting that oocytes from large antral follicles are competent to resume meiosis. These secretory protein-coding genes might serve as biomarkers for certain outcomes of interest.

TFs, which regulate mammalian development, bind to the regulatory regions of genes, forming a complex system that guides the expression of the genome [66]. We found expression of key TFs during antral follicle development, including Homeobox, helix-loop-helix (bHLH), and zf-C2H2, which are essential for transcriptional regulation of developmental processes [67,68]. We also determined that LMX1B, MYRF, and SALL1 are specifically expressed at the small antral follicle, whereas ZNF169 and DLX4 are critical in regulating follicle selection. LMX1B is essential for the maintenance of an appropriately structured actin cytoskeleton in cells [69]. MYRF a potential candidate as an early regulator of gonadal development via upregulation of the transcriptional cofactor CITED2 [70]. SALL1 broadly controls DNA packaging and chromatin remodeling, and its mutation can lead to Müllerian defects [71]. These transcription factors may be critical for maintaining oocyte morphology and chromatin structure in SOs. The hypermethylated gene, ZNF169, encodes a zinc finger DNA-binding protein with unknown function [72]. DLX4 is a member of the DLX family of mammalian homeobox genes. Hara et al. [73] found that DLX4 promoted ovarian tumor through its control of a proangiogenic molecular program involving fibroblast growth factor-2 (FGF-2) and vascular endothelial growth factor (VEGF). It may enable further development of antral follicles by promoting angiogenesis and vascular permeability. TP53 and KLF6 serve as regulatory hubs for many TFs in large antral follicles. TP53 functions as a multitarget transcription factor. Upon cellular stress signals (including DNA damage, hypoxia), p53 is activated to induce growth arrest or cell death, preventing the replication of damaged DNA and the division of genetically altered cells [74,75]. KLF6 regulates growth arrest and can function as a tumor suppressor. The degradation of KLF6 might therefore regulate cell fate decisions between cell cycle arrest and death, depending on the extent of DNA damage [76]. They may play an important role in maintaining the integrity of the maintaining oocyte genome integrity. We found that the regulation of TFs is strongly associated with the follicle development size, and we characterized the interactions between TFs and their target genes to identify the transcriptional regulators of DEGs. The functions of these TFs in oocyte development are only partially known; thus, understanding the role of these TF–DEG pairs in developmental regulation and the underlying molecular mechanisms requires further oocyte studies. 

Our work revealed unique features in the transcriptional machinery and gene signatures of sheep oocytes at different antral follicular sizes. These oocyte-specific genes and TFs may provide valuable clues for future functional studies. First, we identified the gene expression profiles for three antral follicle sizes of oocytes and the functions of stage-specific transcribed genes. Second, we identified key regulatory networks and signaling pathways associated with oocyte development, including those for ribosome biogenesis and a neuroactive ligand receptor. Third, we identified oocyte-specific changes in gene expression that elucidated the regulation of oocyte maturation by maternal transcripts at different sizes. Fourth, we identified subsets of oocyte-specific secretory factors that might serve as biomarkers for specific outcomes of interest. Fifth, we identified stage-specific transcriptional regulators and constructed a regulatory network of core transcriptional regulators and their target genes for the sizes of antral follicle development. Together, these discoveries provide key information on oocyte development and broaden our understanding of sheep folliculogenesis.

## 4. Materials and Methods

### 4.1. Ovarian Stimulation and Oocyte Collection

A total of 12 healthy adult Chinese Hu sheep (1.5–2.5-year-old), procured in April 2023 from the Inner Mongolia JinLai Animal Husbandry Technology Co., Ltd. (Hohhot, Inner Mongolia), were subjected to hormonal treatment as described by Song et al. [77]. The animals received a progestogen-releasing device for 6 d before the initiation of superovulation using FSH soon after normal ovulation, i.e., during the emergence of the first follicular wave. We synchronized estrus in all ewes using the intravaginal progesterone device (CIDR, Pharmacia & Upjohn, Hartwell, Australia) for 6 days. One day prior to the removal of the CIDR, intramuscular injections of 300 IU eCG (Sansheng, Ningbo, China) and 0.12 mg cloprostenol sodium (Sansheng, Ningbo, China) were administered, and then 36 h after removing the CIDR 0.025 mg lecirelin (Sansheng, Ningbo, China) was administered. On the day of ovarian stimulation initiation (D0), each ewe was treated with a new intravaginal progesterone device (CIDR). Thereafter, the ewes received 360 IU of FSH (Sansheng, Ningbo, China) in six decreasing doses (75, 75, 60, 60, 45, and 45 IU) every 12 h (Figure 1A).

Oocyte retrieval was performed as described previously [78]. Briefly, cumulus–oocyte complexes (COCs) were recovered from each donor and classified according to antral follicle size: small (<3 mm; representing early antral follicle), medium (3–6 mm, indicative of the follicle selection), and large (6–10 mm; indicating the follicular dominance). The oocytes and GCs were mechanically stripped using pipet several times. Subsequently, the naked oocytes were collected from small, medium, and large follicles and were labeled as small oocytes (SO), medium oocytes (MO), and large oocytes (LO). The oocytes from each size were separated into different samples (three samples for each size, SO1, SO2, SO3; MO1, MO2; MO3; LO1, LO2, LO3; 10–20 oocytes/each sample) and total RNA was extracted and purified as described previously [79].

### 4.2. Single-Cell RNA Sequencing and Data Analysis

Single-cell full-length cDNA libraries using Smart-seq2 were prepared as previously described [80] using an Illumina Novaseq^TM^ 6000 sequencing platform (Illumina, San Diego, CA, USA). Paired-end reads were processed using an RNA-seq pipeline. The pipeline first performed quality control on the raw reads using Cutadapt (v1.9) and FastQC (v0.11.9), and then aligned the reads to the ovine genome sequence (Ovisaries.Oarv3.1) using the HISAT2 (v2.21) package. An average of 92.5% of the reads per sample were mapped to the reference genome. Next, the mapped reads of each sample were assembled using StringTie (v2.1.6) using the default parameters. All the transcriptomes from all samples were merged to reconstruct a comprehensive transcriptome using gffcompare software (v0.9.8). For the final transcriptome, StringTie and Ballgown were used to estimate the expression levels of all transcripts, and the abundance of the mRNAs was calculated from the fragment per kilobase mapped reads (FPKM).

### 4.3. PCA and Identification of DEGs

We applied the Seurat method to analyze the single-cell data to create the whole clustering profile [81]. Only highly variable marker genes (coefficient of variation > 0.5) were used as inputs for PCA. The marker genes in the PCA plots were plotted using the FeaturePlot function in the Seurat package (v4.0.3). For better accuracy, we complemented the PCA oocyte clustering separately using the FactoMineR (v2.8) package in R (v4.1.0). The multiple *t*-test in the DESeq2 software (v1.43.1) was used to obtain the statistical significance of DEGs in each size (one size versus all other sizes). Only genes with significant *p*-values, an FDR < 0.05, and a log_2_FC-transformed FPKM (fragments per kilobase of exon per million fragments mapped) larger than |2| were considered to be DEGs. The Enhanced Volcano R package was used to visualize the significance of each gene based on its log_2_FC, and the top-ranked 20 markers for each development stage were plotted.

To identify unique gene expression functions and regulatory pathways of oocytes during antral follicle development, we performed GO and KEGG enrichment analysis using the DEGs generated using the R package ClusterProfiler [82]. Gene numbers for terms in GO and KEGG were calculated, and a hyper-geometric test was used to find significantly enriched GO terms (biological process) and KEGG pathways. We defined a term or pathway with an FDR < 0.05 as significantly enriched.

### 4.4. Identification of Stage-Specific DEGs

Only genes with (log_2_FC) greater than |2|, an FDR < 0.05, and expressed in both groups were considered as stage-specific DEGs. GO analysis of stage-specific DEGs was performed with ClusterProfiler. Next, we analyzed stage-specific processes. GO enrichment items for the three samples were obtained based on GSEA (v4.1.0). We selected GSEA terms with *p*-values < 0.05 and listed the top-ranked 10 terms based on the normalized *p*-value enrichment score. We used GSEA and the KEGG pathway enrichment analysis to identify the significantly enriched pathways in oocytes in the transition from medium to large antral. The gene sets with FDR < 0.05 were considered to be enriched.

### 4.5. Identifying Expression Patterns of Oocyte-Specific Genes from DEGs

To characterize the expression patterns of oocyte-specific genes and identify candidate markers for enhancing oocyte developmental competence in vitro, we focused on oocyte-specific functions. The meiosis-related genes and maternal effect genes were downloaded from the HPA database (https://www.proteinatlas.org/, accessed on 13 July 2023). The genes involved in oocyte maturation were downloaded from the MGI database (https://www.informatics.jax.org/, accessed on 13 July 2023) and incorporated into the analysis. Sheep oocyte DEGs were overlapped with genes expressed in human or mouse oocytes to find sheep oocyte candidate genes. The average FPKM value of genes at each time point was imported into STEM software (v1.3.13; School of Computer Science, Carnegie Mellon University) to generate gene expression clusters.

### 4.6. Identifying OSFs at Sequential Antral Follicle Developmental Sizes

To identify candidate biomarkers for predicting oocyte development, we focused on 103 genes encoding oocyte-derived secretory proteins that we downloaded from the HPA database and selected those that overlapped the 9400 sheep oocyte-secreted protein-coding DEGs. These candidate genes were analyzed using the online Search Tool for Recurring Instances of Neighboring Genes (STRING, https://cn.string-db.org//, accessed on 18 July 2023) to construct a PPI network. We set the cut-off as a combined score  >  0.4 based on the number of associations with other genes in the PPI network and visualized the results using CytoScape software (v3.9.1). 

### 4.7. Construction of Transcriptional Regulatory Factor Network in Oocytes

We identified SO vs. LO DEGs (log_2_(FC) > |2| and FDR < 0.05) and selected those that overlapped with 1376 sheep TFs (AnimalTFDB, v4.0) as candidate TFs to construct the network of transcriptional regulators and TF families. To identify the TFs associated with specific biological processes, we used STRING to visualize PPI interaction networks for key TFs. The active interaction sources for our search criteria were limited to Experiments, Databases, Co-expression, Neighborhood, Gene Fusion, and Co-occurrence, with a minimum interaction score set to the medium confidence value of 0.400. We also characterized the TF regulatory network and the differential target genes at different antral follicle sizes. We used the Gene Transcription Regulation Database (GTRD) [83] and the Human Transcription Factor Targets Database (hTFtarget) [84] to find possible targets (within the 9400 DEGs) that could bind the stage-specific TFs. Finally, the TF target regulatory network was visualized using CytoScape software.

### 4.8. Reverse Transcription and qPCR

To analyze the stage-specific gene expression at distinct antral follicle in sheep oocytes, the cDNA of 10 oocytes was synthesized using a reverse transcription system and amplified using the Single Cell Sequence Specific Amplification Kit (Vazyme, Nanjing, China; Cat#P621-01) according to the manufacturer’s instructions [85]. qPCR was conducted with the TB Green^®^ Premix Ex Taq™ II (Tli RNaseH Plus) (Cat#RR820L) on a StepOnePlus Real-Time PCR System (Thermo Fisher Scientific). Ovis aries actin beta (*β-actin*) was used to normalize the data, and gene expression levels were calculated using the comparative cycle threshold (2^−ΔΔCt^) method. The primers were obtained from Sangon Biotech (Shanghai, China), and all qPCR primer pairs are listed in Supplementary Table S17.

### 4.9. Statistical Analysis

The experimental data were analyzed statistically using a two-tailed t-test to compare differences between different sizes with PRISM software (GraphPad 6 Software). An FDR < 0.05 was considered statistically significant. The boxplot represents the median, the first quartile, and the third quartile values.

## 5. Conclusions

In summary, our results highlight the complexity and dynamic nature of oocyte development. We identified stage-specific genes, OSFs, and transcriptional regulators that contribute to the functional and morphological changes that occur during oocyte maturation. Overall, these findings provide insights into the molecular mechanisms that regulate oocyte maturation, with important implications for improving assisted reproductive technologies in domestic animals.

## Data Availability

The original contributions presented in this study are included in the article/Supplementary Material, and further inquiries can be brought to the corresponding author.

## Supplementary Materials

The following supporting information can be downloaded at: https://www.mdpi.com/article/10.3390/ijms25020910/s1.
