# Supplementary material for: Single-Cell Transcriptome Analysis Reveals Development-Specific Networks at Distinct Synchronized Antral Follicle Sizes in Sheep Oocytes
Source: Int J Mol Sci. 2024 Jan 11;25(2):910. doi: 10.3390/ijms25020910 (PMC10815039; doi:10.3390/ijms25020910)
Supplement: Supplementary file 1 [file ijms-25-00910-s001.zip › Figures S1-S5.pdf]

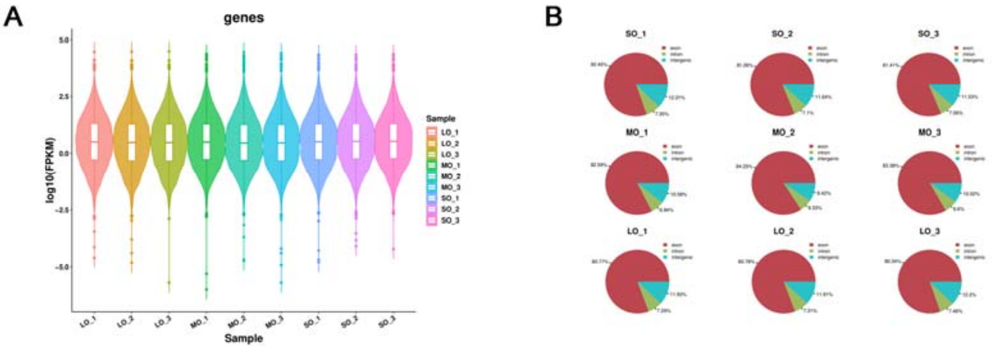

Figure S1. Sample Collection and Quality Control: (A) Violin plot for gene expression level (log10-FPKM) showing he distribution and overall range of the FPKM values in all replicates; (B) Pie chart representing gene coverage distribution

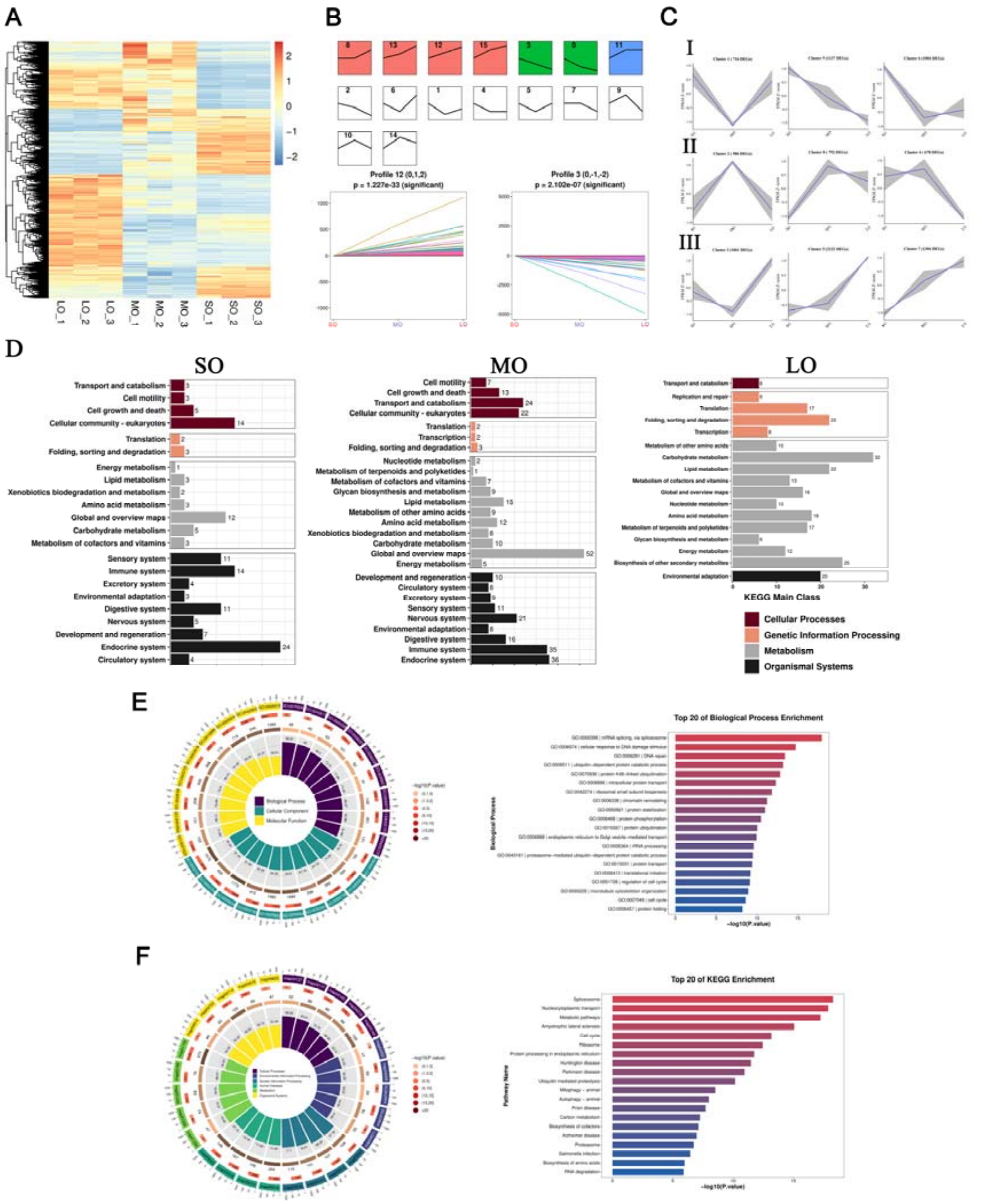

Figure S2. Gene Expression Dynamics and Transcriptional Characteristics during antral follicle development: (A) Hierarchical clustering of 9,400 putative DEGs expression in oocytes during antral follicle development; (B) Above: Clustering analysis of significant DEGs. DEGs were classified into 16 profiles by hierarchical cluster analysis. Each box represents a model expression profile. Below: profiles 12 and profile 3 represent upregulated and downregulated expression during antral follicle development, respectively; (C) K-Means clustering of 9,400 putative DEGs; (D) Significantly enriched KEGG pathways for DEGs in oocytes at three antral follicle stages. (E) Gene ontology LoopCircos of 9,400 putative DEGs; Top 20 GO terms (biological processes) of 9,400 putative DEGs in the oocytes at three stages of antral follicle development. (F) KEGG enrichment LoopCircos of 9,400 putative DEGs; Top 20 KEGG of 9,400 putative DEGs in the oocytes at three stages of antral follicle development

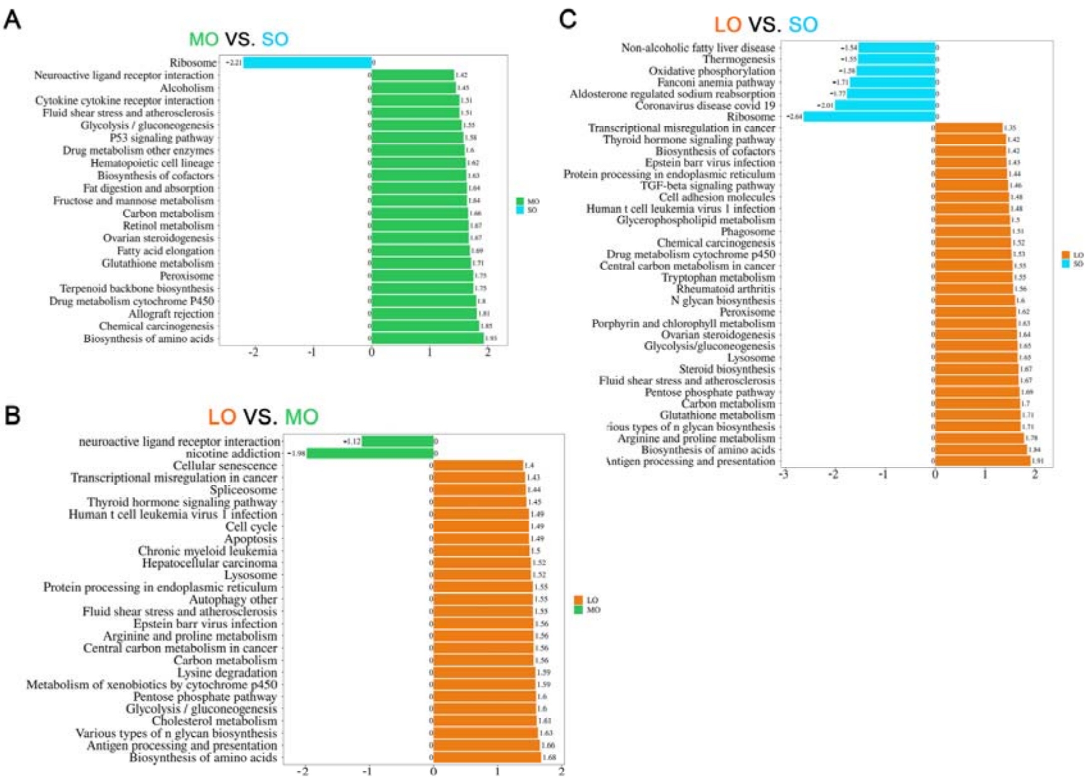

Figure S3. Signaling pathways enriched at each antral follicle stage: (A, B, and C) Representative KEGG terms for upregulated enrichment at each oocyte stage. KEGG terms for each stage are based on the normalized enrichment score (NES). FDR < 0.05;

A

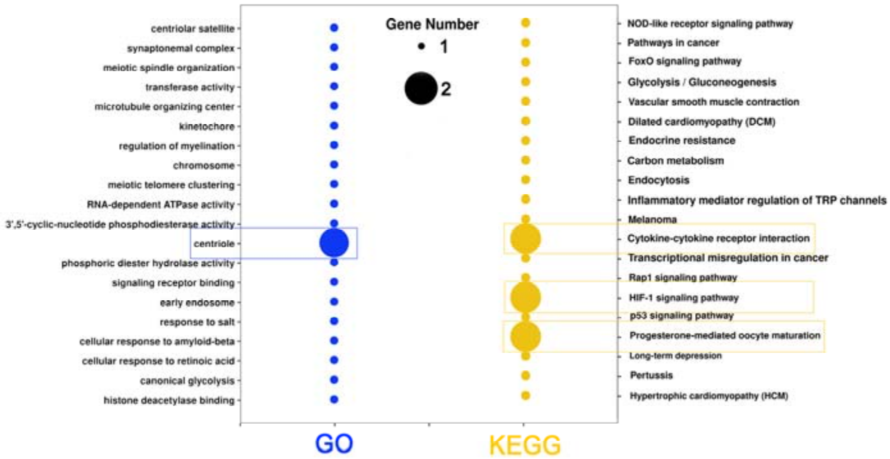

Figure S4. Screening and enrichment analysis of oocyte-specific secretory factors. FDR < 0.05;

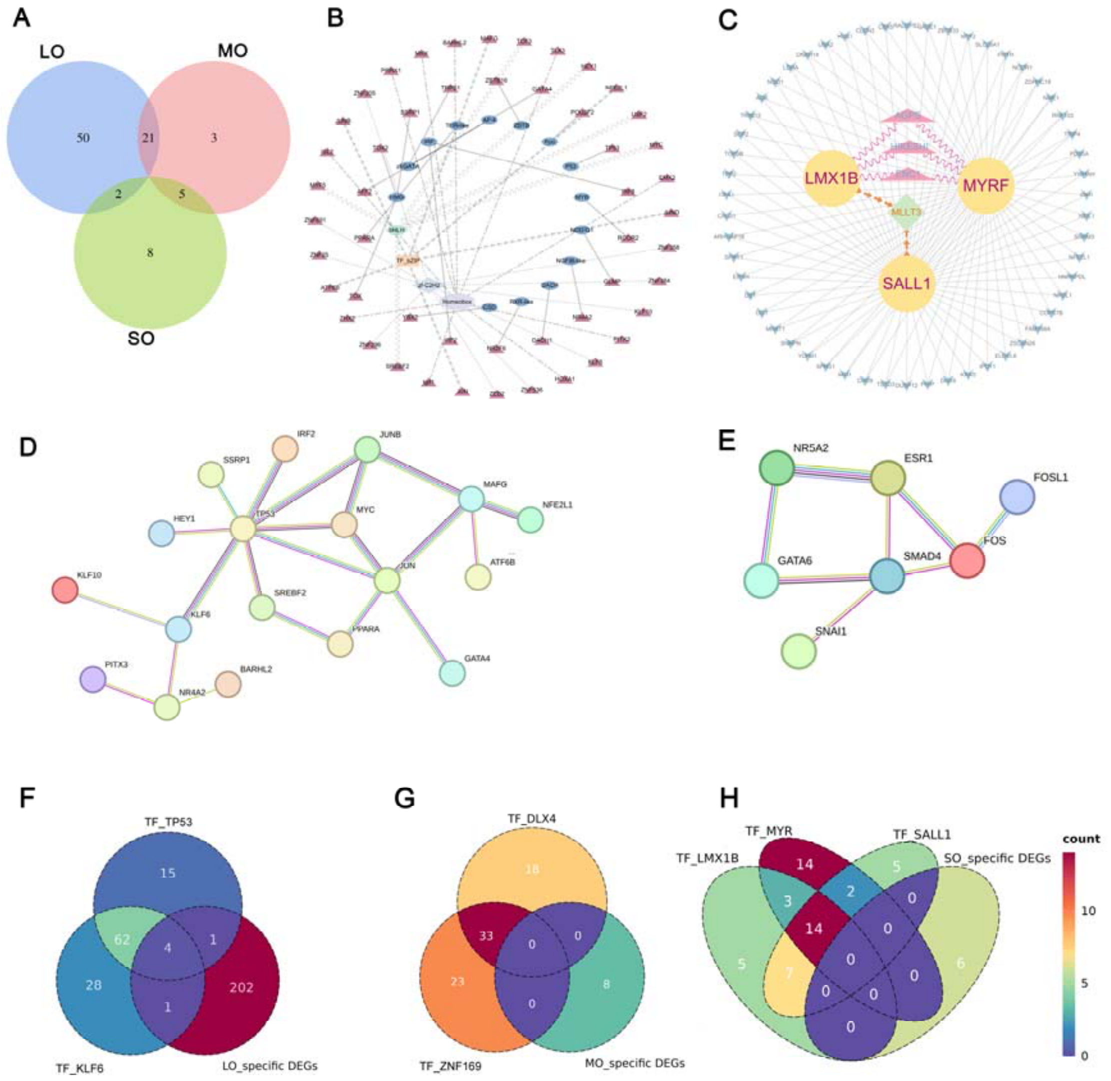

Figure S5. Mapping TF regulatory networks in oocytes from three antral follicle stages**:** (A) Venn diagram showing key TFs among pairwise comparisons of the three antral follicle stages; (B) Visualization by CytoScape of TF regulatory networks in oocytes from the large antral stage. The inner circle shows TF families, whereas the different shapes represent key transcription families, and the outer circle (triangles) shows TFs; (C) CytoScape plots of the TF-DEG regulatory network in small antral follicles. Yellow circles represent TFs, and triangles and diamonds represent co-regulated target genes; (D) The PPI network of unique sheep transcription factors genes: The PPI network of transcription factors in oocytes of large follicle stage; (E) The PPI network of co-expressed in oocytes of medium and large antral follicle stag; (F) Upstream regulators of transcription factors (TP53 and KLF6) in large antral follicles; (G)Upstream regulators of transcription factors (ZNF168 and DLX4) in medium antral follicles; (H) Upstream regulators of transcription factors (LMX1B, MYRF, and SALL1) in small antral follicles.
